# RTS-SLAM: A Trajectory Consistency-Driven Multi-Constraint Dynamic Feature-Rejection Method for Visual SLAM in Dynamic Environments

**DOI:** 10.3390/s26092846

**Published:** 2026-05-02

**Authors:** Huailiang Wang, Qiming Hu, Beicheng Li, Yuhao Geng, Chao Su, Shibo Zhu, Enhui Zheng, Weimin Chen

**Affiliations:** School of Mechanical and Electrical Engineering, China Jiliang University, Hangzhou 310018, China

**Keywords:** dynamic environment, object detection, trajectory consistency constraints, visual simultaneous localization and mapping (SLAM)

## Abstract

Simultaneous Localization and Mapping (SLAM) is a fundamental methodology that underpins autonomous navigation in robotic systems. Conventional approaches perform well in static environments but rely on the assumption of environmental rigidity, which leads to significant accuracy degradation in dynamic environments. To address this challenge, this study presents RTS-SLAM, a real-time semantic visual SLAM system designed for dynamic environments. Based on the ORB-SLAM2 framework, a multi-layer, constraint-driven dynamic feature-rejection strategy is introduced. The proposed approach first removes dynamic features by combining semantic information with geometric constraints. Subsequently, residual dynamic points are eliminated via trajectory-consistency constraint analysis, thereby effectively improving localization accuracy. Furthermore, a dense mapping strategy featuring global sparsification and critical region refinement is proposed. By reducing redundancy in the dense point cloud, the method decreases memory usage while preserving important object geometries. Experimental evaluations on the TUM RGB-D and Bonn datasets indicate that RTS-SLAM reduces the average absolute trajectory error by more than 95% compared with ORB-SLAM2 in dynamic environments. Meanwhile, the system maintains real-time performance and achieves high localization accuracy in dynamic environments.

## 1. Introduction

Within the framework of machine perception, SLAM technology serves as the essential mechanism for facilitating self-location and environmental modeling without pre-existing maps. It relies on the dynamic processing of onboard sensor inputs to derive a robot’s instantaneous pose while simultaneously populating an evolving map of its surroundings. The proliferation of autonomous navigation and immersive computing (AR/VR) has extended SLAM applications far beyond their traditional constraints [1,2,3]. Consequently, these frameworks are increasingly required to maintain robust performance in unpredictable, real-world landscapes characterized by high dynamics and a lack of structured geometry, rather than mere controlled settings. This shift imposes heightened demands on the adaptability and environmental understanding capabilities of SLAM algorithms. Standard visual SLAM frameworks, such as PTAM [4], DSO [5], VINS-Mono [6], and ORB-SLAM3 [7], can achieve accurate trajectory tracking and spatial mapping under the static-scene assumption. However, in scenarios characterized by high dynamics, these systems typically experience a substantial decline in operational effectiveness. Taking the widely used ORB-SLAM2  [8] as an example, when moving objects are present in the scene, its frontend association and backend refinement modules are vulnerable to dynamic features, resulting in significant motion drift, inconsistent map construction, and instability in system tracking.

To optimize the efficacy of visual SLAM frameworks for moving scenes, researchers have introduced various improvement strategies. Early approaches primarily relied on geometric constraints for dynamic feature identification. For instance, DynaSLAM [9] utilized multi-view geometric consistency to distinguish dynamic from static regions, while DS-SLAM [10] combined semantic masks and motion consistency checks for classification. With the advances of deep learning, semantic-based approaches have gained prominence. Ref. [11] applies instance segmentation to detect moving targets like “people” and “vehicles”, then filters out the corresponding feature data. However, while geometry-based methods do not rely on semantic priors and semantic-based approaches leverage semantic information, both suffer from the misclassification of static points as dynamic in scenarios with severe occlusion or significant depth noise. In recent years, hybrid approaches integrating geometric and semantic information have garnered significant attention. Examples include SG-SLAM [12] and YPR-SLAM [13], which rely on geometric constraints supplemented by object detection to counteract dynamic object interference. Despite these advances, existing methods exhibit notable limitations. On the one hand, for objects moving along epipolar lines or lacking semantic cues, their apparent motion closely resembles that of static backgrounds, rendering geometric or semantic discrimination ineffective and leading to missed detections. On the other hand, most approaches fail to adequately analyze the temporal consistency of feature point motion, making it difficult to effectively remove residual dynamic features. Furthermore, in terms of mapping, sparse maps offer higher efficiency but lack geometric information, while dense point cloud maps provide comprehensive information at the cost of substantial memory overhead.

In this study, we propose RTS-SLAM, a visual-semantic SLAM architecture capable of real-time operation in dynamic environments. Building upon the ORB-SLAM2 framework, the proposed strategy employs a hierarchical, constraint-driven approach to filter moving feature points. This approach first integrates semantic prior knowledge with epipolar geometry to achieve coarse-grained dynamic feature removal. Subsequently, by introducing trajectory consistency constraints and analyzing the coherence between feature point 3D motion and camera pose transformations, it removes residual dynamic points, significantly improving the system’s localization accuracy in dynamic environments. Concurrently, a mapping strategy that incorporates global sparsification and critical-region refinement is proposed. This approach substantially reduces point cloud memory consumption while preserving the geometric structure and details of key static regions, thereby enabling low-redundancy and accurate dense map generation in dynamic environments.

This work primarily contributes the following:

(1) Relying on the ORB-SLAM2 architecture, we have designed RTS-SLAM, a real-time RGB-D visual SLAM framework. This system effectively identifies and eliminates interfering features in dynamic environments by integrating multiple constraints: semantic, geometric, and trajectory consistency. Experiments demonstrate that, compared to existing methods, RTS-SLAM yields superior localization precision and operational reliability in dynamic scenes, while simultaneously constructing a structurally coherent dense point cloud map in real time.
(2) The core of this strategy lies in a multi-layer constraint-driven refinement process for dynamic feature elimination. First, it integrates semantic priors from object detection with inter-frame epipolar geometry constraints to perform coarse-grained removal of dynamic features. Subsequently, trajectory consistency constraints are incorporated. By analyzing the consistency between the 3D motion of detected points across consecutive keyframes and camera pose transformations, this approach enables precise removal of residual dynamic points. This strategy effectively eliminates dynamic features while preserving static ones, thereby providing more reliable data for subsequent stages of localization and mapping.
(3) A mapping strategy combining global sparsification with local refinement of critical regions is proposed. This approach first performs global sparsification of point clouds to substantially reduce memory consumption of point cloud maps. Concurrently, to prevent loss of key structural features (such as object contours), locally enhanced and geometrically refined static critical regions are identified based on semantic information. Experiments demonstrate that the proposed methodology effectively preserves the high fidelity of key scene structures while substantially reducing memory consumption, thereby attaining an optimal equilibrium between processing velocity and mapping structural clarity.

The rest of this article is organized as follows. Section 2 reviews relevant work. Methodological details of the system are provided in Section 3. Experimental results are analyzed and discussed in detail within Section 4. Section 5 concludes the paper and presents potential avenues for future work.

## 2. Related Works

In the field of visual SLAM, addressing the interference of moving objects with system stability has become a significant challenge. Conventional visual SLAM frameworks generally rely on the static-environment assumption. However, in dynamic scenarios, moving objects introduce incorrect feature matches, severely affecting both localization accuracy and operational stability. Based on different strategies for handling dynamic features, existing approaches can be divided into three main categories: geometric constraint methods, semantic-informed models, and fusion-based strategies that combine both.

Geometric strategies primarily rely on multi-view consistency or motion-based verification for the identification and rejection of feature points produced by moving objects. Such approaches typically rest upon the fundamental assumption that violates the prescribed geometric constraints dynamically. In pure vision systems, the fundamental matrix is frequently calculated via the RANSAC-coupled seven-point algorithm [14]. Kundu et al. [15] identified dynamic elements by establishing geometric constraints derived from the fundamental matrix. Points are categorized as dynamic in cases where the geometric deviation from their associated epipolar trajectory surpasses a pre-established tolerance. Wang [16] proposed a technique for detecting moving objects by employing analytical mathematical models and geometric constraints. This approach first performs a preliminary assessment of an object’s motion through deep clustering and feature statistics, then introduces inter-frame disparity constraints to further validate its motion state. Additionally, ref. [17] enhanced dynamic semantic features in RGB-D point clouds by utilizing optical flow residuals, thereby achieving motion-static segmentation. Ref. [18] proposes an online removal strategy independent of semantic priors, directly filtering dynamic points using geometric motion features. However, such methods often fail to effectively distinguish in complex, dynamic scenes featuring slow-moving objects, severe occlusions, or sparse textures, and computational efficiency frequently falls short of real-time requirements.

The core concept of semantic information methods lies in utilizing deep learning models to acquire pixel-level semantic priors, actively identifying dynamic regions within images, and eliminating all feature points within these areas. This approach circumvents interference from dynamic features at the data source. For instance, the study in [19] proposed a graph-optimization SLAM framework combining YOLOv5 and Wi-Fi fingerprint sequence matching to improve the reliability of loop closure detection and reduce false loop detection in large-scale environments. DS-SLAM [10] integrates the SegNet [20] semantic segmentation network with optical flow techniques. It eliminates dynamic features via pixel-level moving-object detection and motion-consistency verification, thereby constructing a dense octree semantic map better suited for navigation tasks. Dynamic-DSO [21] employs Mask R-CNN [22] for semantic segmentation, deeply embedding semantic information into a direct SLAM framework to enhance system stability in dynamic environments. Detect-SLAM [23] distinguishes moving and stationary objects in keyframes via object detection, combining motion probability matching with the Randomized Consistency Sampling (RANSAC) algorithm to identify moving targets and eliminate mismatches. By removing feature points from dynamic objects and assigning unique IDs to each, it achieves dense mapping of the target objects. Similarly, Dynamic-SLAM [24] adopts the approach of removing all interest points within detected bounding boxes to suppress disturbances caused by dynamic objects. However, purely semantic dynamic exclusion methods suffer from three inherent limitations. Firstly, their performance is highly dependent on neural network models, creating a significant hurdle to reconciling computational efficiency with segmentation accuracy. Secondly, dynamic recognition capabilities are constrained by predefined semantic categories, resulting in insufficient generalization ability for unknown dynamic targets beyond the training set. Furthermore, in regions where static and dynamic elements coexist, the absence of geometric constraints often leads to the erroneous rejection of static features that should be retained.

Over the past several years, the trajectory of dynamic SLAM studies has evolved from relying on isolated information sources to developing hybrid frameworks that combine semantic and geometric constraints, significantly enhancing localization accuracy and operational efficiency in dynamic environments. Within this trend, multiple fusion strategies have been proposed. For instance, Chang et al. [11] combined YOLACT instance segmentation with optical flow compensation to establish a two-stage dynamic point filtering mechanism, though it remains limited under complex motion conditions. YOLO-SLAM [25] employs YOLOv3 [26] to detect potential moving targets, utilizing RANSAC to eliminate feature points within dynamic regions. OVD-SLAM  [27] further integrates YOLOv5 semantic segmentation, optical flow, and depth information. By distinguishing foreground from background and averaging multi-frame reprojection errors, it restores static points on moving object surfaces, thereby adapting to non-rigid motion and low-dynamic scenes. Zhu et al. [28] proposes an improved YOLOv9S-based SLAM framework integrated with ORB-SLAM2, where enhanced object detection, epipolar geometry constraints, and depth separation strategies are employed to remove dynamic features. DIO-SLAM [29] employs YOLACT instance segmentation, dynamically classifying rigid masks using optical flow residuals and consistency criteria, while compensating for misdetections by leveraging temporal frame propagation techniques. While effective under typical dynamic conditions, these hybrid approaches still encounter constraints when faced with sophisticated movement patterns. Specifically, techniques utilizing optical flow consistency often struggle to identify non-rigid motion or objects moving along epipolar lines, which may lead to detection failures and the erroneous removal of static points.

To mitigate the detrimental impact of environmental dynamics on SLAM reliability, we introduce a hierarchical framework driven by multiple constraints to eliminate dynamic feature points. Initially, the proposed strategy incorporates semantic information alongside geometric constraints to perform preliminary removal of dynamic features. Subsequently, trajectory consistency constraints are introduced for refined filtering, enabling accurate detection and removal of potential dynamic outliers. The proposed strategy significantly improves the precision of feature removal while enhancing overall system efficiency.

## 3. System Overview

This chapter provides an overview of the RTS-SLAM system structure, which is divided into five components. First, it outlines the overall architecture of the system along with its basic operational sequence. Second, the target identification module is introduced, which provides semantic feature inputs to the system. Subsequently, the dynamic feature classification criteria based on epipolar geometry constraints are described. Third, a refinement strategy for dynamic feature rejection based on trajectory consistency constraints is presented. Finally, the dense map construction method used in the system is detailed.

### 3.1. System Framework

**Figure 1:**
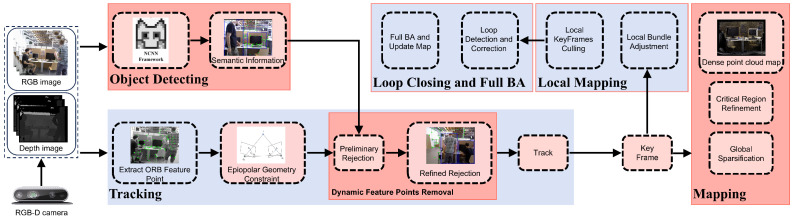
Structural layout of the RTS-SLAM framework. The portions marked in blue represent the legacy ORB-SLAM2 scheme, while the crimson sections emphasize the modular expansions introduced in this work.

Derived from ORB-SLAM2 [8], the RTS-SLAM framework augments the standard execution threads—tracking, local mapping, and loop detection—by integrating two parallel processing units for target recognition and dense mapping. By leveraging a multi-threaded parallel architecture, the framework preserves precise self-localization capabilities while significantly enhancing operational efficiency. The holistic design layout of this platform is visualized in Figure 1. Once initialized, the system maintains synchronized ingestion of both color frames and depth maps sourced via the RGB-D camera. The tracking thread executes ORB feature extraction on the incoming image frames, providing the fundamental input for the real-time localization algorithm. Subsequently, the system integrates semantic priors provided by the object detection thread to perform preliminary motion classification on the feature points. Utilizing epipolar line geometry constraints, it completes an initial round of coarse-motion feature rejection based on semantic and geometric fusion. Building upon this, trajectory consistency constraints are incorporated. By analyzing whether the three-dimensional motion trajectories of feature points across consecutive key frames align with camera pose transformations, the system achieves refined rejection of residual motion features. Within the dense mapping thread, we employ a mapping strategy combining global sparsification with local refinement in critical regions. This approach significantly reduces point cloud storage redundancy through global sparsification while enhancing local point density and refining geometric structures in structurally critical static areas. Ultimately, the system generates dense point cloud representations in dynamic settings, thereby balancing global coherence with high-fidelity local attributes.

### 3.2. Object Detection

Dynamic environments pose significant interference to visual SLAM systems. To address this, the system incorporates a lightweight object detection module. This module leverages the NCNN architecture, which is specifically tailored for mobile deployment to facilitate efficient neural network inference. Developed entirely in native C++ without external library dependencies, its highly parallelized architecture ensures real-time operational efficiency on resource-constrained platforms.

In model selection, the system employs the lightweight Single Shot MultiBox Detector (SSD) [30] architecture. This framework achieves both object localization and classification through a single forward pass, with relatively straightforward post-processing, thereby significantly reducing computational latency. To further optimize efficiency, the SSD backbone utilizes the lightweight MobileNetV3 [31]. This network employs separable convolutions and neural architecture search techniques to substantially reduce parameter count and computational overhead while maintaining detection accuracy. The complete model leverages prior knowledge acquired from the Pascal VOC [32] corpus, facilitating the robust identification of prevalent non-static entities, including human figures and various automobiles.

Contrastive to the computationally intensive semantic segmentation networks utilized in [9,10,11,33], the SSD detector integrated into our framework generates solely bounding boxes and category labels. This strategic simplification bypasses the pixel-level mask generation process, markedly curtailing processing delays. Consequently, as the cornerstone of mobile robot state estimation, the SLAM system’s real-time responsiveness becomes a critical prerequisite for the successful implementation of high-level missions.

### 3.3. Epipolar Constraints

In dynamic visual SLAM systems, accurate identification of static references within dynamic environments is essential to mitigate estimation drift and achieve high pose accuracy. While deep learning–based object detection methods provide semantic prior information for identifying potential moving objects such as pedestrians and vehicles, semantic information alone cannot determine their actual motion state at the observation moment. For instance, seated pedestrians or stationary vehicles detected as ‘people’ or ‘vehicles’ may contain feature points that remain static, contributing to camera motion estimation. Indiscriminately discarding all feature points associated with such semantic categories would not only discard valuable static observations but also compromise system stability in environments with many stationary structures. To further determine feature point motion states based on semantic prior knowledge, this paper introduces epipolar geometry constraints for preliminary dynamic feature screening between adjacent frames. Initially, the system establishes ORB feature correspondences across consecutive frames using a pyramidal iterative Lucas–Kanade (LK) optical flow scheme, while filtering out unreliable matches in peripheral image regions. Based on these correspondences, the inter-frame fundamental matrix is estimated from stable matches using the RANSAC-based seven-point method. By leveraging the geometric constraints defined by this fundamental matrix, the distance between each feature point and its corresponding epipolar line is computed. If this value remains below a preset threshold, the point is classified as static and retained; otherwise, it is flagged as dynamic and subsequently eliminated.

**Figure 2:**
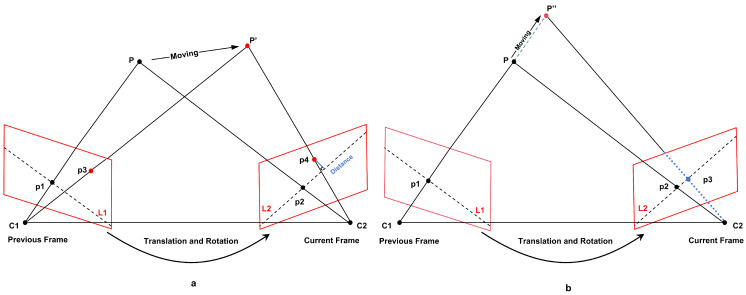
Epipolar geometry constraints. (**a**) The general case of dynamic features subject to epipolar geometric constraints. (**b**) The degenerate scenario where dynamic features undergo collinear motion relative to the epipolar line direction.

As illustrated in Figure 2, the pinhole camera model characterizes the geometric correspondence between the same spatial point *P* across adjacent image frames. $C_{1}$ and $C_{2}$ denote the optical centers of the cameras, respectively. The spatial point *P* is mapped to feature points $P_{1}$ and $P_{2}$, respectively, within the preceding and current frame-relative camera coordinate systems. The plane defined by $C_{1}$, $C_{2}$, and *P* is designated as the epipolar plane; its intersections with the image planes of the two frames yield the epipolar lines $L_{1}$ and $L_{2}$ (illustrated by red dashed segments), while $P^{\prime }$ and $P^{\prime \prime }$ denote the reprojected coordinates of the shifted map point. Feature points $P_{1}$ and $P_{2}$ are expressed in homogeneous coordinates as follows:

(1) $$P_{1}=\left[\begin{array}{c} x_{1}\\ y_{1}\\ 1 \end{array}\right],\;P_{2}=\left[\begin{array}{c} x_{2}\\ y_{2}\\ 1 \end{array}\right].$$

where *x* and *y* constitute the pixel coordinates of the feature point. The fundamental matrix *F* is then utilized to determine the epipolar line $L_{2}$ within the current frame:

(2) $$L_{2}=FP_{1}=F\left[\begin{array}{c} x_{1}\\ y_{1}\\ 1 \end{array}\right]=\left[\begin{array}{c} X\\ Y\\ Z \end{array}\right].$$

where *X*, *Y*, and *Z* constitute the elements of the line vector. Based on the principles in [15], the epipolar constraint is defined by

(3) $$P_{2}^{T}FP_{1}=P_{2}^{T}L_{2}=0.$$

For feature point $P_{2}$, the distance *D* to its corresponding epipolar line is given by

(4) $$D=\frac{\left|P_{2}^{T}FP_{1}\right|}{\sqrt{X^{2}+Y^{2}}}.$$

From the Formula (4), ideally, feature point $P_{2}$ falls precisely onto the epipolar line $L_{2}$ in the current frame; consequently, the calculated distance *D* should be equivalent to zero. In practical operating scenarios, however, due to interference from diverse noise sources, feature points frequently deviate from the theoretical epipolar line, in which case the value of *D* exceeds zero while remaining within the allowable tolerance margin. As shown in Figure 2a, the movement of point *P* to $P^{\prime }$ causes the spatial deviation of the projected point $P_{4}$ to exceed the allowable tolerance margin, leading to its categorization as a dynamic feature and its immediate removal. Nevertheless, relying solely on the geometric properties of the polar line is generally insufficient to address all dynamic scenarios. In Figure 2b, geometric degeneration occurs when processing dynamic points moving along the polar line: if spatial point *P* undergoes radial translation along the linear extension of $C_{1}P$, upon attaining position $P^{\prime \prime }$, its corresponding projection $P_{3}$ within the polar plane remains situated on epipolar line $L_{2}$, leading to a null displacement *D* when calculated via Formula (4). This leads to the point being misclassified as static. Therefore, this paper introduces a strategy for eliminating trajectory consistency constraints. By analyzing the consistency between the 3D motion paths of observations and camera pose updates, this constraint effectively identifies dynamic points moving along the polar line, thereby achieving a more refined elimination of residual dynamic features.

### 3.4. Trajectory Consistency Constraint Rejection Strategy

To address the limitations of geometric constraints and semantic detection in removing dynamic feature points, trajectory consistency constraints analyze the 3D motion patterns of features across successive frames. The fundamental principle relies on the spatial alignment between feature displacement and camera ego-motion. While the apparent motion of stationary landmarks follows the camera motion, descriptors from moving objects exhibit significant motion inconsistencies. Therefore, by evaluating the alignment between feature trajectories and camera motion, dynamic and static elements can be effectively differentiated. In practical implementation, matched keypoints from consecutive frames are first transformed into the 3D camera coordinate system using depth information. Subsequently, the spatial displacement of each feature point across two frames and the ego-motion displacement of the camera are computed. By comparing these two displacement magnitudes, the motion state of each point can be determined. If the difference between the feature displacement and camera motion exceeds a predefined threshold, the point is classified as dynamic and removed; otherwise, it is retained as static.

In the proposed methodology, suppose that *N* pairs of correlated feature points are identified between two consecutive frames, which can be formally represented as

(5) $$(p_{t-1}^{i},p_{t}^{i}),\;i=1,2,\ldots ,N.$$

Let $p_{t-1}^{i}={\left[x_{t-1}^{i},y_{t-1}^{i}\right]}^{T}$ and $p_{t}^{i}={\left[x_{t}^{i},y_{t}^{i}\right]}^{T}$ denote the image-plane coordinates of the *i*-th feature across consecutive frames. For the purpose of mapping these pixel locations into the camera coordinate system, they are reformulated into a homogeneous coordinate form

(6) $$P_{t-1}^{i}=\left[\begin{array}{c} x_{t-1}^{i}\\ y_{t-1}^{i}\\ 1 \end{array}\right],\;P_{t}^{i}=\left[\begin{array}{c} x_{t}^{i}\\ y_{t}^{i}\\ 1 \end{array}\right].$$

By leveraging the information from the depth map, the depth values associated with these two frames are represented as

(7) $$D_{t-1}^{i-1}\left(x_{t-1}^{i},y_{t-1}^{i}\right),\;D_{t}^{i}\left(x_{t}^{i},y_{t}^{i}\right).$$

Through the camera’s intrinsic matrix:

(8) $$K=\left[\begin{array}{ccc} f_{x} & 0 & c_{x}\\ 0 & f_{y} & c_{y}\\ 0 & 0 & 1 \end{array}\right].$$

The variables $f_{x}$ and $f_{y}$ correspond to the orthogonal focal lengths, with $\left(c_{x},c_{y}\right)$ marking the principal point where the optical axis aligns with the image plane’s center. Utilizing these intrinsic parameters, feature points are back-projected into the 3D camera coordinate space as follows:

(9) $$P_{t-1}^{i}=D_{t-1}^{i}K^{-1}P_{t}^{i}=\left[\begin{array}{c} \left(x_{t-1}^{i}-c_{x}\right)\frac{D_{t-1}^{i}}{f_{x}}\\ \left(y_{t-1}^{i}-c_{y}\right)\frac{D_{t-1}^{i}}{f_{y}}\\ D_{t-1}^{i} \end{array}\right],\;P_{t}^{i}=D_{t}^{i}K^{-1}P_{t}^{i}=\left[\begin{array}{c} \left(x_{t}^{i}-c_{x}\right)\frac{D_{t}^{i}}{f_{x}}\\ \left(y_{t}^{i}-c_{y}\right)\frac{D_{t}^{i}}{f_{y}}\\ D_{t}^{i} \end{array}\right].$$

Subsequently, the geometric displacement $d_{pc}$ of each feature point in 3D space is evaluated across the sequential observations.

(10) $$d_{pc}^{i}=∥P_{t}^{i}-P_{t-1}^{i}∥.$$

The translation component *t* is extracted directly from the camera’s estimated pose transition matrix $T_{t,t-1}=\left[\begin{array}{cc} R & t\\ 0 & 1 \end{array}\right]$, where the magnitude of this vector represents the camera’s own motion displacement $d_{cam}$:

(11) $$d_{cam}=∥t_{t,t-1}∥.$$

In static scenes, this displacement is primarily caused by the camera’s own motion, and its magnitude should satisfy:

(12) $$∥d_{pc}^{i}∥\approx ∥d_{cam}∥.$$

Given that feature points exhibit significant variations in their projected scale on the imaging plane at different depths, their sensitivity to pixel changes varies across their three-dimensional displacements. Applying a uniform fixed threshold directly may lead to misclassification of minute displacements of dynamic points within close-range scenes as static points, or cause failure to correctly identify a close-range dynamic point due to its displacement exceeding the threshold without triggering detection. Therefore, to achieve discrimination that better aligns with true geometric relationships, trajectory consistency constraints must incorporate depth information of feature points. This study introduces a depth-dependent tolerance threshold σ into the trajectory consistency constraint to adjust the acceptable displacement range at different depths.

(13) $$\sigma =\alpha +\beta \cdot \min(d_{t-1}^{i},d_{t}^{i}).$$

In this formulation (13), α denotes the baseline tolerance, primarily employed to compensate for camera intrinsic residuals, pixel quantization noise, and minor stochastic fluctuations during feature extraction. β is a scaling factor reflecting the influence of depth, designed to balance the heteroscedasticity of depth measurement uncertainty that intensifies with increasing distance. Although $D^{i}$ and $d^{i}$ both originate from the same depth map and encapsulate depth information, they fulfill distinct roles: $D^{i}$ serves as a scale factor for 3D back-projection in coordinate transformation, whereas $d^{i}$ acts as a depth scalar to modulate the trajectory consistency threshold during motion identification.

Potential dynamic outliers are isolated by assessing the absolute deviation between feature displacement and the camera’s ego-motion relative to the threshold σ.

(14) $$\Delta d^{i}=\left|d_{pc}^{i}-d_{cam}\right|,Status=\left\{\begin{array}{cc} \mathrm{Dynamic}, & \mathrm{if}\;\Delta d^{i}>\sigma .\\ \mathrm{Static}, & \mathrm{if}\;\Delta d^{i}\leq \sigma . \end{array}\right.$$

The tactical approach to rejecting dynamic features is detailed in Algorithm 1. This method first performs preliminary screening of feature points based on extreme line constraints to obtain candidate static features. Subsequently, it combines depth information and introduces trajectory-consistency constraints to conduct secondary, refined elimination of the remaining dynamic features.
**Algorithm 1** Dynamic Feature Rejection Strategy**Input:** 
Previous frame’s feature points, $P_{1}$; Current frame’s feature points, $P_{2}$; Previous frame’s static feature points, $P_{1sta}$; Current frame’s static feature points, $P_{2sta}$; Previous frame, $F_{1}$; Current frame, $F_{2}$; Previous depth map, $D_{1}$; Current depth map, $D_{2}$; Camera intrinsic matrix, *K*; Dynamic object culling threshold, ξ; tolerance threshold, σ; Number of feature points threshold, $N_{th}$;**Output:** 
Set of identified non-static points in the current frame, $S_{dynamic}$;  1: $P_{2}=\mathrm{EstimateOpticalFlow}\left(F_{1},F_{2}\right)$;  2: Filter out potential moving features located near image boundaries;  3: **for** each matched pair $P_{1sta},P_{2sta}$ whithin $P_{1},P_{2}$ **do**  4:    **if** Point $P_{1}$ is situated outside semantic dynamic regions **then**  5:      Append $P_{1},P_{2}$ to $P_{1sta},P_{2sta}$  6:    **end if**  7:    Feature ++  8: **end for**  9: **if** Feature $>N_{th}$ **then**10:   Compute Fundamental Matrix *F* via RANSAC on $\left\{P_{1sta},P_{2sta}\right\}$;11:**else if** Feature $\leq N_{th}$ and DynamicFlag is active **then**12:   Compute Fundamental Matrix *F* via RANSAC on $\left\{P_{1},P_{2}\right\}$;13:**end if**14:**for** each matched pair $p_{1},p_{2}$ in $P_{1},P_{2}$ **do**15:   **if** Distance to epipolar line $d_{epi}\left(p_{1},p_{2},F\right)>\xi$ **then**16:     Append $P_{2}$ to $S_{dynamic}$;17:   **else if** Valid depth values exist in both $D_{1}$ and $D_{2}$ **then**18:     Recover 3D spatial coordinates $P_{1}$ and $P_{2}$ using *D* and *K*;19:     Calculate individual point displacement: $d_{pc}=∥P_{2}-P_{1}∥$;20:     Extract camera ego-motion displacement from pose: $d_{cam}=∥t∥$;21:     **if** Displacement discrepancy $|d_{pc}-d_{cam}|>\sigma$ **then**22:        Record $p_{2}$ into the dynamic feature set $S_{dynamic}$;23:     **end if**24:   **end if**25:**end for**

### 3.5. Dense Mapping

Within the traditional ORB-SLAM2 [8] framework, maps are primarily constructed based on sparse feature points. While this approach ensures stable pose tracking, it struggles to accurately capture the fine-grained geometric structure of real-world scenes. These limitations are notably intensified in dynamic scenarios, where the limited descriptive power of sparse data, coupled with its susceptibility to disturbances stemming from mobile entities, significantly impairs the integrity and utility of the generated mapping output. To address these deficiencies, RTS-SLAM maintains sparse mapping for efficient pose estimation while concurrently integrating depth information to construct dense point clouds. This strategy substantially bolsters the system’s capability for comprehensive geometric representation of the environment.

In large-scale SLAM scenarios, dense mapping significantly increases the number of points in the global point cloud. This imposes a dual challenge: it extends the duration of the dense mapping phase, thereby reducing the execution speed of the overall SLAM system, and introduces substantial memory requirements. Such high memory demands create a risk of memory overflow, which consequently compromises system stability. Typically, research in this field prioritizes the detailed representation of salient objects within dense point clouds, while assigning lower importance to background elements such as walls and tabletops.

Based on the aforementioned scenarios and requirements, RTS-SLAM proposes a dense-mapping strategy that integrates global sparsification and key-region refinement. As described in Algorithm 2. Global sparsification refers to applying voxel grid filtering to the depth point cloud of the keyframe. This process downsamples the point cloud by retaining representative points within each voxel cell.

The mathematical formulation of voxel grid filtering is given as follows. The original point cloud generated from the depth map $D_{k}$ of the keyframe $F_{k}$ is defined as:

(15) $$P_{k}=\left\{p_{i}={\left(x_{i},y_{i},z_{i}\right)}^{T}|i=1,2,\ldots ,N\right\}.$$

where *N* denotes the number of points in the original point cloud. Given the voxel resolution $r_{v}$, the 3D space is partitioned into a set of cubic voxels with side length $r_{v}$, where each voxel corresponds to a cubic region in the Euclidean space.For each point $p_{i}={\left(x_{i},y_{i},z_{i}\right)}^{T}$, its corresponding voxel index is defined as

(16) $$v_{i}=\left(\left\lfloor \frac{x_{i}}{r_{v}}\right\rfloor ,\left\lfloor \frac{y_{i}}{r_{v}}\right\rfloor ,\left\lfloor \frac{z_{i}}{r_{v}}\right\rfloor \right),\;v_{i}\in Z^{3}.$$

where ⌊·⌋ denotes the floor operation, and $v_{i}$ represents the discrete voxel grid coordinate. All points sharing the same voxel index *v* are grouped into a voxel cell, defined as

(17) $$V_{v}=\left\{p_{i}\in P_{k}|v_{i}=v\right\}.$$

For each non-empty voxel (i.e., $|V_{v}|>0$), the representative point is computed as the centroid of all points within the voxel:

(18) $${\bar{p}}_{v}=\frac{1}{|V_{v}|}\sum_{p_{i}\in V_{v}}p_{i}.$$

Thus, the downsampled point cloud after voxel grid filtering can be expressed as

(19) $$P_{k}^{\;s}=\left\{{\bar{p}}_{v}|V_{v}\neq \emptyset \right\}.$$

where $|V_{v}|$ denotes the number of points contained in voxel *v*, and $P_{k}^{\;s}$ represents the downsampled point cloud after voxel grid filtering. The computational complexity of voxel grid filtering is approximately linear with respect to the number of input points, as each point is assigned to a voxel once, and centroid computation is performed only over occupied voxels.

The remaining points are subsequently transformed into the world coordinate system and integrated into the global point cloud map.

The key region refinement method is based on global downsampling. By utilizing image information and object detection results from several common frames closest to the keyframe, only the point clouds of the detected static key objects are transformed into the world coordinate system and saved as the global point cloud map, thereby enhancing the details of the target objects in the dense map.
**Algorithm 2** Global Sparsification and Key-Region Refinement**Input:** 
Keyframe $F_{k}$; Neighboring frames $F_{i}$; Depth maps $D_{k},D_{i}$; Camera intrinsics matrix *K*; Camera poses $T_{wc}^{k},T_{wc}^{i}$; Voxel resolution $r_{v}$; Temporal window size $N_{w}$;**Output:** 
Updated global dense map *P*  1: Generate depth point cloud $P_{k}$ from $D_{k}$ using *K*  2: Apply voxel grid filtering with resolution $r_{v}$ to obtain sparse point cloud $P_{k}^{\;s}$  3: Transform $P_{k}^{\;s}$ into world coordinates using $T_{wc}^{k}$  4: Insert filtered points into global map *P*  5: Detect object bounding boxes $B_{k}$ in keyframe $F_{k}$  6: **for** each neighboring frame $F_{i}$ **do**  7:    **if** $\left|i-k\right|\leq N_{w}$ **then**  8:      Identify corresponding object regions in frame $F_{i}$  9:      Extract depth points from $D_{i}$ within these regions10:     Remove dynamic points using multi-constraint filtering11:     Transform remaining points to world coordinates via $T_{wc}^{i}$12:     Merge refined points into *P*13:   **end if**14:**end for**15:**return** *P*

**Figure 3:**
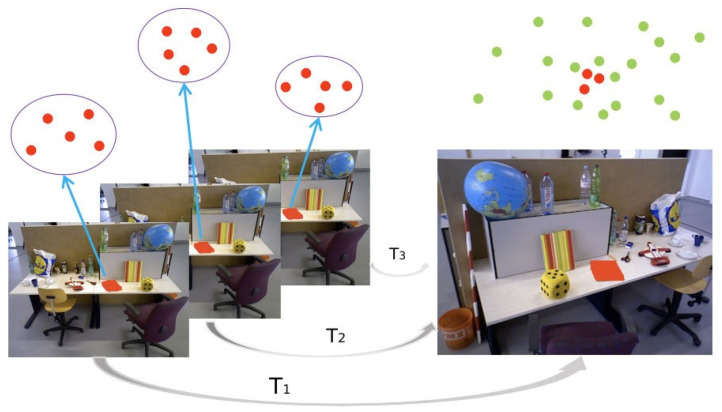
Schematic diagram of the key-region refinement process for targeted objects.

Figure 3 illustrates the key region refinement method. In this example, a book on the desk is defined as the “key object”, with its target region localized by a 2D bounding box generated by the detection network. The image on the right represents the keyframe, where the overlaying points constitute the point cloud in the camera coordinate system, obtained by downsampling the keyframe’s depth map; the red points specifically denote the point cloud of the book. The multiple images on the left are frames preceding the keyframe, with the red points in each frame representing the book’s point cloud derived from the corresponding pixels in their respective camera coordinate systems. By integrating point clouds from both the left and right frames into the global map, the geometric completeness of the target object in the dense map is enhanced.

## 4. Experimental Results

This section experimentally evaluates and analyses the proposed RTS-SLAM system across four dimensions. Initial quantitative evaluations focus on tracking precision, utilizing the TUM RGB-D [34] and Bonn [35] datasets as benchmarks. Subsequently, the performance advantages of our integrated fusion mechanism are verified via ablation experiments. Following this, the mean processing durations are compared across relevant algorithms to appraise the real-time viability of the system. Furthermore, the intuitive quality and structural integrity of the generated dense maps are showcased through visual reconstructions. To ensure statistical reliability and minimize stochastic errors, 20 independent trials were conducted for each data sequence. The final performance metrics were derived by calculating the trimmed mean, specifically by discarding the extreme outliers (maximum and minimum values). All computational tasks were performed on a workstation running Ubuntu 20.04, powered by an Intel i5-13400F CPU, an NVIDIA GeForce RTX 5060 GPU, and 16 GB of memory.

### 4.1. Performance Evaluation on TUM RGB-D Dataset

The Technical University of Munich’s Computer Vision Group provides the TUM RGB-D dataset [34], which functions as a comprehensive and authoritative benchmark for the validation of diverse SLAM methodologies. By providing synchronized color and depth streams from RGB-D sensors, the dataset encompasses diverse indoor environments and introduces significant dynamic challenges, such as pedestrian motion and manual gestures. To verify the effectiveness of RTS-SLAM in complex settings, five representative sequences were adopted for empirical study: fr3_walking_xyz, fr3_walking_rpy, fr3_walking_half, fr3_walking_static, and fr3_sitting_static. The initial four sequences represent high-dynamic scenarios characterized by intense human activity and frequent occlusions. Conversely, the final sequence features low-dynamic elements, serving to evaluate the system’s steady-state performance and adaptability in near-static surroundings.

To rigorously quantify positioning precision, two standard evaluation metrics were utilized: Absolute Trajectory Error (ATE) and Relative Pose Error (RPE). ATE assesses global consistency by determining the translational deviation between estimated and ground-truth trajectories. In contrast, RPE is partitioned into RTE (Relative Translational Error) and RRE (Relative Rotational Error) to evaluate local drift between sequential frames. System accuracy was further validated through four statistical metrics: RMSE, mean, median, and S.D. To verify the effectiveness of RTS-SLAM, we benchmarked it against the original ORB-SLAM2 across five dynamic sequences, as detailed in Table 1, Table 2 and Table 3.

**Figure 4:**
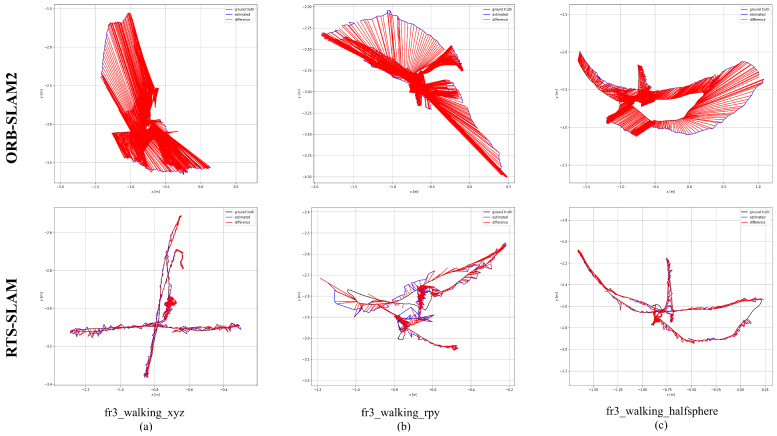
ATE assessment for RTS-SLAM and ORB-SLAM2 evaluating five distinct data sequences: (**a**) fr3_walking_xyz. (**b**) fr3_walking_rpy. (**c**) fr3_walking_halfsphere.

**Figure 5:**
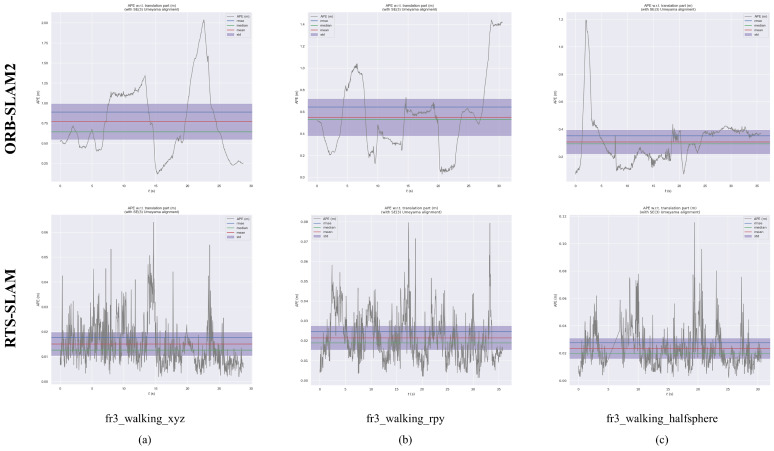
ATE results for RTS-SLAM versus ORB-SLAM2 across the TUM RGB-D benchmark sequences: (**a**) fr3_walking_xyz. (**b**) fr3_walking_rpy. (**c**) fr3_walking_halfsphere.

As shown in Table 1, Table 2 and Table 3, RTS-SLAM exhibits substantial enhancements over ORB-SLAM2 across all evaluation metrics within highly dynamic environments. The maximum enhancements in RMSE, Mean, Median, and S.D. reached 98.19%, 98.21%, 98.14%, and 98.13%, respectively. In low-dynamic sequences, RTS-SLAM also demonstrates outstanding performance, with improvements of 32.44%, 33.26%, 34.59%, and 31.41%, respectively, across the aforementioned metrics. The absolute trajectory error (ATE) comparisons between ORB-SLAM2 and RTS-SLAM for the five evaluated sequences are further visualized in Figure 4 and Figure 5. The ground-truth trajectories are depicted by grey dashed lines, while the estimated poses are traced by blue curves. The red regions highlight the positional discrepancies. Under identical dynamic constraints, ORB-SLAM2 suffers from substantial trajectory estimation inaccuracies. Conversely, RTS-SLAM yields estimated paths that more closely match the ground-truth data. Such outcomes confirm the superior positioning performance of the proposed framework within highly dynamic environments.

To achieve a comprehensive appraisal of RTS-SLAM, we conduct a performance comparison against several leading frameworks. The evaluation involves referencing established methods including ORB-SLAM3 [7], SG-SLAM [12], RDS-SLAM [36], and COEB-SLAM [37]. Regarding the performance results illustrated in Table 4. Specifically, for the fr3_sitting_static sequence, the environment can be considered weakly dynamic or near-static due to minimal human pose variations. Under such conditions, methods relying on semantic segmentation, such as DS-SLAM, can reliably preserve static regions while suppressing unstable observations caused by minor human movements, which leads to slightly better localization accuracy. For the fr3_walking_xyz sequence, YOLO-SLAM achieves slightly better performance due to its object detection module, which can directly identify dominant dynamic objects such as humans and reduce their influence on pose estimation. Since the dynamic elements in this sequence mainly belong to detectable semantic categories, the object-level filtering mechanism becomes particularly effective. In contrast, sequences such as fr3_walking_static, fr3_walking_rpy, and fr3_walking_half contain more complex motion patterns and irregular dynamic disturbances. In such cases, semantic detection alone may fail to capture all dynamic elements. RTS-SLAM addresses this challenge through a multi-constraint dynamic feature rejection strategy, which integrates semantic cues, geometric constraints, and trajectory consistency analysis to remove feature points that violate global motion consistency. By filtering unreliable observations caused by dynamic objects while preserving reliable static features, RTS-SLAM maintains stable pose estimation and achieves more robust localization performance in highly dynamic environments.

To further clarify the differences between the proposed method and existing dynamic SLAM approaches, Table 5 summarizes the main technical characteristics of several representative methods, including the utilization of semantic information, geometric constraints, motion consistency, and their strategies for handling dynamic objects.

### 4.2. Performance Evaluation on Bonn RGB-D Dataset

Originating from the University of Bonn in 2019, the Bonn RGB-D dataset [35] provides 24 RGB-D sequences characterized by significant dynamic disturbances. This benchmark is frequently utilized for assessing how visual SLAM frameworks operate within intricate, non-static settings. To test the generalization and efficacy of our method in dynamic contexts, we selected nine representative sequences from this dataset for experimentation. These encompass diverse motion patterns, such as multiple individuals moving freely within an indoor setting in the “crowd” sequence, a person relocating an object in “moving no box”, the continuous camera tracking of a slow-moving individual in “person tracking”, and complex synchronous dynamic patterns of multiple individuals repeatedly jumping in unison and in the same direction in “synchronous”. Given its more intense and varied dynamic activity relative to the TUM RGB-D dataset, the Bonn dataset presents a more rigorous challenge for SLAM systems.

Regarding the performance metrics illustrated in Table 6, RTS-SLAM shows a slight deficit compared to SG-SLAM in the “moving no box1” sequence. This performance gap stems from the continuous presence of moving objects, such as boxes being transported, which frequently cause transient occlusions and swift modifications to the local structure of the scene. While RTS-SLAM emphasizes long-term consistency, SG-SLAM utilizes the frame-by-frame integration of semantic and geometric data for dynamic suppression, allowing for a more immediate response to such rapid environmental transitions. In both “synchronous” sequences, RTS-SLAM demonstrated inferior localization accuracy compared to YOLO-SLAM. This disparity is mainly due to the tightly integrated framework of YOLO-SLAM, which combines geometric priors with object recognition. By utilizing depth disparity alongside the RANSAC technique to isolate non-static elements, YOLO-SLAM successfully mitigates the pose drift triggered by coordinated movements. Conversely, the trajectory consistency constraints employed by RTS-SLAM are prone to misclassifying highly synchronized dynamic point clouds as static background, thereby introducing estimation errors. Nevertheless, in view of the comprehensive outcomes presented in Table 6, the proposed framework consistently achieves superior results throughout the majority of test sequences. This highlights its capacity for reliable localization within varied dynamic surroundings.

### 4.3. Ablation Experiment

To analyze the effectiveness of the unified framework incorporating geometric, semantic, and trajectory consistency constraints within dynamic environments, this paper conducted a series of ablation experiments. These experiments compared the effectiveness of algorithms relying solely on semantic information, solely on geometric information, and on the integrated approach. Specifically, RTS-SLAM (S) relies exclusively on semantic information for dynamic feature removal. RTS-SLAM (G) relies exclusively on polar line geometric constraints to identify dynamic points. RTS-SLAM (S+G) integrates semantic information with geometric constraints to facilitate the identification and removal of dynamic outliers. RTS-SLAM (S+G+T) implements a comprehensive dynamic feature rejection strategy by integrating semantic detection, polar line geometry constraints, and trajectory consistency constraints. A detailed performance comparison across different modules is showcased in Table 7.

**Figure 6:**
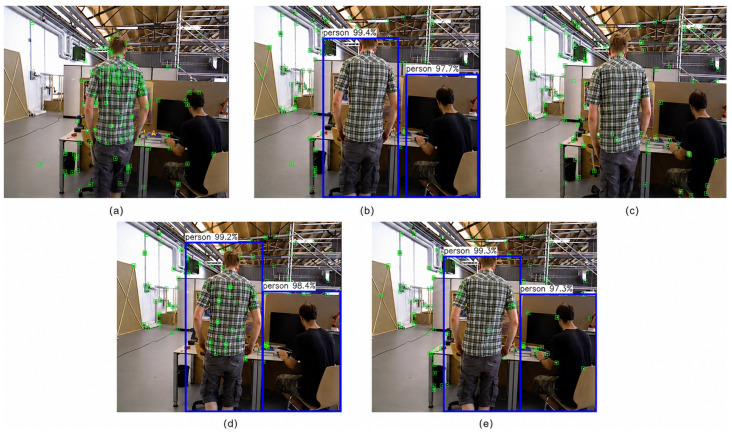
Visualization of dynamic feature rejection performance across different modules. (**a**) ORB-SLAM2. (**b**) RTS-SLAM (S). (**c**) RTS-SLAM (G). (**d**) RTS-SLAM (S+G). (**e**) RTS-SLAM (S+G+T).

Figure 6 provides a comparative analysis of feature point extraction and dynamic content removal results across distinct combinations of constraints. Figure 6a shows the feature points extracted by ORB-SLAM2, revealing that it has scarcely processed dynamic regions (such as pedestrians), retaining a significant number of dynamic feature points. Figure 6b presents results using semantic information alone. While feature points within human regions are effectively removed, some erroneous retention persists in dynamically active areas with insignificant semantic content (e.g., edges of rapidly moving objects). Figure 6c demonstrates results using only polar line geometry constraints. This approach erroneously removed some feature points in static regions (e.g., tabletop and monitor edges) while failing to fully identify and filter certain dynamic targets (e.g., walking pedestrians). By contrast, the strategy in Figure 6d, which fuses semantic information with polar line geometry constraints, achieves a more balanced outcome: effectively removing dynamic feature points while preserving key features in the static background. Furthermore, Figure 6e illustrates the comprehensive framework incorporating trajectory consistency constraints. Building upon the fusion of semantic and geometric data, this strategy further validates the feature points by evaluating their motion coherence across consecutive frames. This effectively suppresses residual dynamic interference, resulting in a more stable and refined final feature distribution. The quantitative results presented in Table 7, coupled with the qualitative findings from Figure 6, further validate the efficacy of the proposed multi-constraint fusion approach.

### 4.4. Time Analysis

To evaluate the operational feasibility of RTS-SLAM, we assessed its computational overhead by measuring the mean execution time per frame. The results were then benchmarked against current state-of-the-art SLAM solutions to verify their real-time capabilities. A summary of the real-time performance metrics is presented in Table 8, the baseline ORB-SLAM2 system achieved the shortest frame processing time due to its omission of semantic processing modules. Systems employing pixel-level semantic segmentation (such as DS-SLAM and Dyna-SLAM), while enabling finer dynamic object segmentation, introduced significant processing latency owing to their higher computational complexity, rendering them unsuitable for stringent real-time requirements. YOLO-SLAM, based on the YOLO object detection algorithm, offers advantages in detection accuracy. However, its operational speed is constrained by the algorithmic architecture and hardware limitations, posing challenges for real-time performance. In contrast, RTS-SLAM achieves an average frame processing time of merely 23.8 ms, maintaining high real-time responsiveness while realizing superior processing efficiency.

### 4.5. RTS-SLAM Dense Mapping Experiment

**Figure 7:**
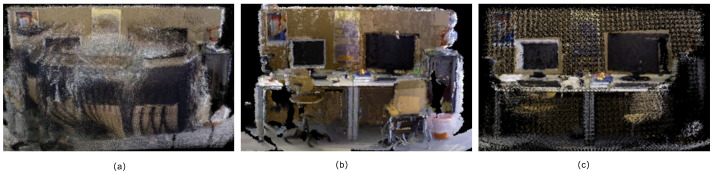
Comparison of dense mapping results. (**a**) Dense map without dynamic object removal. (**b**) Dense map after removing dynamic objects. (**c**) Dense map generated using the proposed global sparsification and key-region refinement strategy.

The dense reconstruction capabilities of RTS-SLAM in highly dynamic scenarios were extensively verified through a series of experiments on the TUM RGB-D dataset. Figure 7 illustrates the point cloud reconstruction performance comparison of the proposed mapping strategy at different processing stages. In the baseline configuration lacking dynamic handling (Figure 7a), potential moving objects within the scene introduce a significant volume of mismatched point clouds during motion. This results in pronounced ghosting and structural distortion in the reconstruction, which significantly undermines the structural fidelity and overall consistency of the resulting dense map. Upon implementing dynamic point cloud removal (Figure 7b), anomalous point clouds generated by potential moving objects are effectively filtered out. The geometric contours of static scene structures become markedly clearer, fundamentally improving the reconstruction quality. Further integrating global sparsification and critical region refinement (Figure 7c) enables the system to dynamically adjust point cloud density globally and locally based on semantic target distribution and depth information, while retaining key object details and optimizing resource utilization. As illustrated in Figure 7c, high-density point clouds are concentrated in critical target areas (such as the monitor, keyboard, and books on the desk), while sparse distribution is maintained in other regions, preserving essential structure while avoiding redundancy. Experimental statistics indicate that the dense point cloud in Figure 7b comprises 1,569,892 points, whereas Figure 7c contains only 307,200 points. This represents an approximately 80.4% reduction in total point cloud volume, significantly lowering demands on memory and computational resources. Since each point requires memory to store its spatial coordinates and associated attributes, the overall memory consumption is approximately proportional to the number of stored points. Therefore, reducing redundant points directly decreases the memory footprint of the dense map. To further evaluate the efficiency of the proposed dense mapping strategy, Table 9 presents a comparison of point cloud size, reduction rate, memory consumption, and mapping time under different strategies. Furthermore, many operations in dense mapping, such as neighborhood search, point fusion, and map updates, have computational costs that depend on the size of the point cloud. By reducing the point cloud size while preserving important regions, the proposed strategy can effectively reduce the computational overhead of the mapping process.

## 5. Conclusions

To tackle the challenges of complex dynamic settings, this research introduces RTS-SLAM, a real-time semantic visual SLAM framework. Built upon the ORB-SLAM2 architecture, the system implements a hierarchical dynamic feature suppression strategy: it first fuses semantic priors with epipolar geometry constraints for coarse-grained suppression, followed by trajectory consistency constraints to remove residual dynamic noise across consecutive frames. This methodology effectively mitigates interference from moving entities in camera ego-motion estimation, significantly improving positioning stability and metric accuracy in highly dynamic scenarios. Furthermore, during the dense-mapping phase, RTS-SLAM employs global sparsification and localized critical-region refinement. By prioritizing the geometric fidelity of salient objects while substantially reducing point cloud redundancy, the system reduces memory overhead and facilitates dense map construction for large-scale scenes.

Further refinements are required for the proposed system in future research. At the algorithmic level, efforts will focus on enhancing the generalization capability of semantic models and optimizing the spatial density of the constructed maps. At the hardware level, the integration of active sensing modalities, such as LiDAR, will be explored to compensate for the inherent limitations of pure vision solutions in depth estimation within unstructured environments.

## Figures and Tables

**Table 1:** ATE performance of RTS-SLAM versus ORB-SLAM2 on TUM RGB-D sequences (unit: m).

Sequences	ORB-SLAM2				RTS-SLAM (Ours)				Improvements			
	RMSE	Mean	Median	S.D.	RMSE	Mean	Median	S.D.	RMSE (%)	Mean (%)	Median (%)	S.D. (%)
fr3_walking_xyz	0.8882	0.7704	0.6420	0.4420	0.0161	0.0138	0.0119	0.0083	98.19	98.21	98.14	98.13
fr3_walking_static	0.3455	0.3146	0.2606	0.1428	0.0069	0.0061	0.0055	0.0033	98.00	98.06	97.90	97.68
fr3_walking_rpy	0.6435	0.5498	0.5303	0.3344	0.0254	0.0199	0.0156	0.0158	96.05	96.38	97.06	95.26
fr3_walking_half	0.3531	0.3090	0.2954	0.1710	0.0245	0.0208	0.0171	0.0129	93.06	93.25	94.20	92.48
fr3_sitting_static	0.0101	0.0094	0.0090	0.0056	0.0068	0.0063	0.0059	0.0038	32.44	33.26	34.59	31.41

**Table 2:** Relative translational error (RPE) metrics for the proposed RTS-SLAM versus ORB-SLAM2 across the TUM RGB-D benchmark (unit: m/s).

Sequences	ORB-SLAM2				RTS-SLAM (Ours)				Improvements			
	RMSE	Mean	Median	S.D.	RMSE	Mean	Median	S.D.	RMSE (%)	Mean (%)	Median (%)	S.D. (%)
fr3_walking_xyz	0.3408	0.2801	0.2495	0.2340	0.0127	0.0105	0.0088	0.0072	96.27	96.25	96.46	96.94
fr3_walking_static	0.2468	0.0947	0.0635	0.1122	0.0075	0.0064	0.0056	0.0039	96.95	93.22	91.15	96.49
fr3_walking_rpy	0.3636	0.2261	0.1587	0.1643	0.0209	0.0153	0.0120	0.0142	94.26	93.23	92.46	91.37
fr3_walking_half	0.2246	0.1689	0.1260	0.1480	0.0165	0.0129	0.0098	0.0103	92.66	92.39	92.25	93.02
fr3_sitting_static	0.0149	0.0133	0.0108	0.0097	0.0083	0.0070	0.0059	0.0045	44.04	47.04	45.15	53.75

**Table 3:** Relative rotational error (RPE) metrics for the proposed RTS-SLAM versus ORB-SLAM2 across the TUM RGB-D benchmark (unit: °/s).

Sequences	ORB-SLAM2				RTS-SLAM (Ours)				Improvements			
	RMSE	Mean	Median	S.D.	RMSE	Mean	Median	S.D.	RMSE (%)	Mean (%)	Median (%)	S.D. (%)
fr3_walking_xyz	7.0159	5.2133	4.4186	4.0487	0.4664	0.3988	0.3296	0.2418	93.35	92.35	92.54	94.03
fr3_walking_static	3.6274	1.6689	0.5533	3.4836	0.2263	0.2053	0.1924	0.0953	93.76	87.70	65.23	97.26
fr3_walking_rpy	6.8102	4.9095	2.0211	4.0618	0.9630	0.7040	0.4995	0.6571	85.86	85.66	75.29	83.82
fr3_walking_half	7.0227	4.2485	1.7517	6.5915	0.7818	0.6661	0.5566	0.4092	88.87	84.32	68.23	93.79
fr3_sitting_static	0.3830	0.3150	0.3189	0.2214	0.2750	0.2399	0.2235	0.1345	28.20	23.85	29.93	39.28

**Table 4:** ATE performance of the proposed RTS-SLAM compared with other dynamic SLAM algorithms on the TUM RGB-D benchmark (unit: m).

Seq.	ORB-SLAM3		DS-SLAM		SG-SLAM		COEB-SLAM		RDS-SLAM		YOLO-SLAM		DynaSLAM		RTS-SLAM (Ours)	
	RMSE	S.D.	RMSE	S.D.	RMSE	S.D.	RMSE	S.D.	RMSE	S.D.	RMSE	S.D.	RMSE	S.D.	RMSE	S.D.
fr3_w_xyz	0.7012	0.3018	0.0247	0.0161	0.0180	0.0094	0.0182	0.0094	0.0571	0.0229	**0.0146**	**0.0070**	0.0156	0.0079	0.0161	0.0083
fr3_w_static	0.4218	0.2246	0.0081	0.0067	0.0081	0.0036	0.0074	0.0032	0.0206	0.0120	0.0073	0.0035	0.0079	0.0043	**0.0069**	**0.0033**
fr3_w_rpy	0.8656	0.4526	0.4442	0.2350	0.0341	0.0213	0.0342	0.0231	0.1604	0.0874	0.2164	0.1001	0.0325	0.0194	**0.0254**	**0.0158**
fr3_w_half	0.6280	0.2926	0.0303	0.0283	0.0313	0.0194	0.0303	0.0158	0.0807	0.0454	0.0283	0.0138	0.0261	0.0123	**0.0245**	**0.0129**
fr3_s_static	0.0090	0.0043	**0.0065**	**0.0033**	0.0072	0.0032	0.0074	0.0036	0.0084	0.0043	0.0066	0.0033	0.0067	0.0028	0.0068	0.0038
Mean	0.5251	0.2551	0.1027	0.0578	0.0197	0.0114	0.0195	0.0110	0.0654	0.0344	0.0546	0.0255	0.0177	0.0093	**0.0159**	**0.0088**

**Note:** Bold values indicate the best performance in each sequence among all compared methods.

**Table 5:** Comparison of representative dynamic SLAM methods in terms of dynamic feature handling strategies.

Method	Semantic	Geometric	Motion Consistency	Dynamic Object Handling Strategy
ORB-SLAM3	No	Yes	Yes	Assumes a static environment and does not explicitly handle dynamic objects
DS-SLAM	Yes (semantic segmentation)	Yes	No	Removes feature points on detected dynamic objects using semantic masks
SG-SLAM	Yes	Yes	Yes	Combines semantic segmentation and geometric constraints to filter dynamic points
COEB-SLAM	Yes (object-level)	Yes	No	Uses object-level constraints and bounding boxes to suppress dynamic regions
RDS-SLAM	Yes	Yes	No	Integrates semantic information with depth-based geometric consistency to suppress dynamic features
YOLO-SLAM	Yes (object detection)	Yes	Yes	Combines object detection with geometric verification to filter dynamic features
DynaSLAM	Yes (Mask R-CNN)	Yes	No	Segments dynamic objects and removes associated feature points
**RTS-SLAM (Ours)**	**Yes**	**Yes**	**Yes**	**Multi-constraint dynamic feature rejection using semantic cues, geometric constraints, and trajectory consistency**

**Note:** Bold values indicate the proposed method (RTS-SLAM (Ours)) for clearer visual comparison with other methods.

**Table 6:** ATE assessment of the proposed RTS-SLAM versus contemporary state-of-the-art algorithms using the Bonn benchmark (unit: m).

Seq.	ORB-SLAM2		YOLO-SLAM		SG-SLAM		RTS-SLAM (Ours)	
	RMSE	S.D.	RMSE	S.D.	RMSE	S.D.	RMSE	S.D.
crowd1	0.8632	0.6284	0.033	-	0.0234	0.0143	**0.0202**	**0.0113**
crowd2	1.3573	1.2071	0.423	-	0.0584	0.0406	**0.0246**	**0.0143**
crowd3	1.0772	1.0070	0.069	-	0.0319	0.0219	**0.0308**	**0.0181**
moving_no_box1	0.1174	0.0935	0.027	-	**0.0192**	**0.0081**	0.0211	0.0122
moving_no_box2	0.1142	0.0973	0.035	-	0.0299	**0.0030**	**0.0267**	0.0104
person_tracking1	0.7959	0.7090	0.157	-	0.0400	0.0139	**0.0382**	**0.0115**
person_tracking2	1.0679	0.9590	0.037	-	0.0376	0.0154	**0.0251**	**0.0131**
synchronous1	1.1411	0.9884	0.014	-	0.3229	0.1824	**0.0823**	**0.0564**
synchronous2	1.4069	1.3201	**0.007**	-	0.0164	0.0126	0.0138	**0.0088**

**Note:** Bold values indicate the best performance in each sequence among all compared methods.

**Table 7:** ATE assessment for distinct system modules evaluated on the TUM RGB-D benchmark (unit: m).

Seq.	S		G		S+G		S+G+T	
	RMSE	S.D.	RMSE	S.D.	RMSE	S.D.	RMSE	S.D.
fr3_w_xyz	0.0181	0.0097	0.0216	0.0099	0.0170	0.0089	**0.0161**	**0.0083**
fr3_w_static	0.0094	0.0053	0.0102	0.0054	0.0077	0.0036	**0.0069**	**0.0038**
fr3_w_rpy	0.0359	0.0225	0.2350	0.1485	0.0310	0.0190	**0.0254**	**0.0158**
fr3_w_half	0.0763	0.0578	0.0400	0.0255	0.0306	0.0176	**0.0245**	**0.0129**
fr3_s_static	0.0083	0.0042	0.0099	0.0046	0.0079	0.0039	**0.0068**	**0.0038**
Runtime (ms)	21.3		18.5		22.6		23.8	

**Note:** Bold values indicate the best performance in each sequence among all ablation settings.

**Table 8:** Real-time performance evaluation.

Systems	Average Processing Time per Frame (ms)	Hardware Platform
YOLO-SLAM	696.09	Intel Core i5-4288U CPU
ORB-SLAM2	16.4	Intel Core i5-13400F CPU, NVIDIA GeForce RTX 5060 GPU
SG-SLAM	39.8	Intel Core i5-13400F CPU, NVIDIA GeForce RTX 5060 GPU
COEB-SLAM	48.7	Intel Core i5-13400F CPU, NVIDIA GeForce RTX 5060 GPU
RTS-SLAM (ours)	23.8	Intel Core i5-13400F CPU, NVIDIA GeForce RTX 5060 GPU

**Table 9:** Quantitative comparison of dense mapping efficiency under different strategies.

Method	Points	Reduction (%)	Memory (MB)	Mapping Time (ms)
Original Dense Map	1,569,892	–	120	41.0
Voxel Grid Filter	520,000	66.9	40	20.8
RTS-SLAM (Ours)	307,200	80.4	24	18.7

## Data Availability

The original contributions presented in this study are included in the article. Further inquiries can be directed to the corresponding author.
